# Temperature-Induced Structural Changes in Muscle Proteins from Giant Squid (*Dosidicus gigas*) Mantle: FT-IR, Circular Dichroism, and FE-SEM Analysis

**DOI:** 10.3390/foods14172922

**Published:** 2025-08-22

**Authors:** Miguel A. León-Heredia, Enrique Marquez-Rios, Francisco Cadena-Cadena, Hisila Santacruz-Ortega, Ignacio Alfredo Rivero-Espejel, Nathaly Montoya-Camacho, Iván J. Tolano-Villaverde

**Affiliations:** 1 Department of Food Technology Engineering, Universidad Estatal de Sonora, Campus Hermosillo, Ley Federal del Trabajo s/n, Hermosillo 83100, SON, Mexico; miguelleonheredia@gmail.com; 2 Department of Food Research and Graduate Studies, Universidad de Sonora, Boulevard Luis Encinas y Rosales, Hermosillo 83000, SON, Mexico; 3 National Technological Institute from Mexico, Valle del Yaqui Campus, Road 600, San Ignacio Río Muerto 85276, SON, Mexico; fcadena.cadena@itvy.edu.mx; 4 Department of Research in Polymers and Materials, Universidad de Sonora, Boulevard Luis Encinas y Rosales, Hermosillo 83000, Son, Mexico; hisila.santacruz@unison.mx; 5 Graduate and Research Center in Chemistry, Instituto Tecnológico de Tijuana, Tijuana 22414, BC, Mexico; irivero@tectijuana.mx; 6 Department of Chemical Biological Sciences, Universidad de Sonora, Boulevard Luis Encinas y Rosales, Hermosillo 83000, SON, Mexico; nathaly.montoya@unison.mx

**Keywords:** giant squid proteins, thermal denaturation, secondary structure, morphological characteristics, functional properties

## Abstract

The giant squid (*Dosidicus gigas*) is an abundant marine species with high protein content, making it a promising resource for the food and biomaterial industries. This study aimed to investigate the effect of temperature (25–100 °C) on the structural changes in sarcoplasmic, myofibrillar, and stromal proteins isolated from squid mantle. Fourier-transform infrared spectroscopy (FT-IR) and circular dichroism (CD) were employed to monitor modifications in secondary structure, while field emission scanning electron microscopy (FE-SEM) was used to examine morphological characteristics. The FT-IR analysis revealed temperature-induced transitions in amide I, II, and A bands, indicating unfolding and aggregation processes, particularly in myofibrillar and stromal proteins. CD results confirmed a loss of α-helix content and an increase in β-sheet structures with rising temperature, especially above 60 °C, suggesting progressive denaturation. FE-SEM micrographs illustrated clear morphological differences: sarcoplasmic proteins displayed smooth, amorphous structures; myofibrillar proteins exhibited fibrous, porous networks; and stromal proteins presented dense and layered morphologies. These findings highlight the different thermal sensitivities and structural behaviors of squid muscle proteins and provide insight into their potential functional applications in thermally processed foods and bio-based materials.

## 1. Introduction

The giant squid (*Dosidicus gigas*) is a widely distributed cephalopod and an abundant marine resource along the Pacific coast, ranging from California to Chile [1]. Its muscle, characterized by being white, lean, and free of scales, bones, and pigments, due to the presence of hemocyanin rather than hemoglobin, offers several advantages over traditional fish species. These features, along with its low cost and ease of processing, make *D. gigas* a promising candidate for the development of protein concentrates and functional food ingredients [2]. Previous studies have focused mainly on the myofibrillar proteins of *D. gigas* due to their key role in gelation and emulsification. However, increasing attention has also been directed toward its sarcoplasmic and stromal proteins, especially collagen, given their unique functional and structural properties. Based on their solubility, the muscle proteins of *D. gigas* can be fractionated into approximately 30% sarcoplasmic proteins (water-soluble), 65% myofibrillar proteins (soluble in high ionic strength), and 5% stromal proteins (insoluble at any ionic strength) [3].

Each protein fraction exhibits distinct technological properties. Sarcoplasmic proteins have been shown to enhance foaming capacity, while myofibrillar proteins exhibit strong gelling and emulsifying capabilities, although sometimes inferior to those of fish proteins. Stromal proteins, due to their poor solubility, have been less studied from a functional perspective, but their rigid and fibrous nature suggests potential for applications in biomaterials, especially when combined with other biopolymers such as chitosan [4]. Among the various factors influencing protein functionality, temperature plays a central role. Most food-processing techniques involve thermal treatments that can induce protein denaturation, aggregation, or gelation phenomena directly related to the structural integrity of proteins. Therefore, understanding the thermal behavior of squid muscle proteins is essential for optimizing their use in food and material applications [5].

Most thermal analyses on squid have been performed using differential scanning calorimetry (DSC) or thermogravimetric analysis (TGA), which evaluate protein denaturation temperatures and the energy required for such transitions, primarily by measuring enthalpy change (ΔH). Additionally, other thermal evaluations include rheological methods, such as viscosity and dynamic oscillatory measurements (G′ and G″), as a function of temperature [6]. However, these techniques do not allow for an objective assessment of the conformational and structural changes occurring, nor do they identify the specific functional groups involved at the evaluated temperatures. Consequently, methodologies such as circular dichroism and infrared spectroscopy are valuable tools for analyzing protein secondary structure and identifying the functional groups available or participating in colloidal systems [7,8,9]. Furthermore, a more detailed understanding of protein structure and morphology enables the establishment of more suitable processing conditions and, therefore, broader applications in various fields. Understanding the thermal behavior of squid muscle proteins is thus essential for optimizing their use in food and material applications, including the enhancement of physicochemical properties of food, encapsulation of bioactive compounds, and development of biocompounds, among others.

The aim of this study was to isolate and characterize the sarcoplasmic, myofibrillar, and stromal proteins from *D. gigas* mantle and evaluate their structural and morphological changes under increasing temperatures (25–95 °C). To achieve this, we employed Fourier-transform infrared spectroscopy (FT-IR), circular dichroism (CD), and field emission scanning electron microscopy (FE-SEM) to assess their secondary structure and microstructural behavior in response to thermal stress.

## 2. Materials and Methods

### 2.1. Sampling

Jumbo squid (*Dosidicus gigas*) was harvested off the coast of Kino Bay, Mexico. Ten specimens were de-headed and degutted on site, and washed with freshwater. Mantles (experimental samples) were bagged and placed in alternating layers of ice–squid–ice in a portable cooler, and transported to the laboratory. The time elapsed between capture and reaching the laboratory did not exceed 12 h. 

### 2.2. Ethical Statement

The present study did not involve any live animal experiments. Specimens of Dosidicus gigas (jumbo squid) used in this research were obtained post-mortem from artisanal fishery landings in Kino Bay, Sonora, Mexico. The animals were already deceased at the time of collection, and no procedures were performed on live animals. Therefore, ethical approval by an institutional review board or animal ethics committee was not required. According to current Mexican legislation (e.g., NOM-033-SAG/ZOO-2014 and guidelines for scientific research in aquatic organisms), the post-mortem use of invertebrate marine species not listed as endangered or protected does not require prior ethical approval. Thus, this study complies with national and institutional ethical policies and with the ethical standards required by the journal Foods.

### 2.3. Sarcoplasmic Protein Isolation

The fractionation of squid mantle proteins was carried out using the procedure described by Hashimoto et al. [10]. For this, the frozen squid mantle was thawed at 4–5 °C for 12 h, minced, and combined with cold distilled water (≤4 °C) in a 1:3 ratio. The mixture was then homogenized for 1 min at 1000 rpm using a tissue homogenizer (Wisd; WiseTis HG-15D; Witeg, Wertheim, Germany). The resulting homogenate was centrifuged in a refrigerated centrifuge at 12,000× *g* for 20 min at 4 °C (Thermo Scientific; Sorvall Biofuge Stratos; Waltham, MA, USA). The supernatant obtained was regarded as the sarcoplasmic protein (SP) fraction, and the precipitate was used for myofibrillar extraction.

### 2.4. Myofibrillar Isolation

The previous precipitate was resuspended in 100 mL of phosphate buffer (15.6 mM Na_2_HPO_4_, 0.45 M KCl, pH 7.5, and I = 0.5) and then centrifuged at 12,000× *g* for 20 min at 4 °C (Thermo Scientific; Sorvall Biofuge Stratos; Waltham, MA, USA). The obtained precipitate was stored to use in stromal protein recovery, while the supernatant was homogenized with 300 mL of distilled water (4 °C) and then centrifuged at 15,500× *g* for 30 min at 4 °C. This precipitate was considered the myofibrillar fraction [11].

### 2.5. Stromal Protein Isolation

The first precipitate obtained during myofibrillar extraction was homogenized at 4 °C with 300 mL of 0.1 M NaOH for 12 h. After alkaline treatment, the sample was thoroughly washed with distilled water to remove excess alkali and then centrifuged at 10,000× *g* for 15 min at 4 °C. The resulting precipitate was considered the stromal protein fraction [11]. 

### 2.6. Fourier Transform Infrared Spectrometers (FT-IR)

FT-IR spectra were obtained using 1 mg of freeze-dried mantle protein, sarcoplasmic, myofibrillar, and stromal fractions, along with 5 μL of distilled water (for assessing molecular flexibility). A temperature sweep from 25 to 100 °C was performed, and a reading was taken every 5 °C in an infrared spectrometer (Frontier FT-IR, Perkin Elmer, Waltham, MA, USA) with attenuated total reflection (ATR) at a scan interval from 500 to 4000 cm^−1^. The results were expressed as absorbance versus wavenumber [6].

### 2.7. Circular Dichroism (CD)

CD spectra were obtained using a spectropolarimeter (J-815, JASCO, Tokyo, Japan) with a peltier accessory (PFD-425S/15, JASCO, Tokyo, Japan). The preparation of the working solution was made from a stock solution of 1 g/mL in phosphate buffer (15.6 mM Na_2_HPO_4_, pH 7.0), and concentrations of all solutions were adjusted to 0.0215 mg/mL. This process was carried out by constant stirring for 24 h at a temperature of 25 °C. The scanning wavelengths from 163 to 400 nm were registered. Results were expressed as mean residual molar ellipticity (θ). The secondary structure percentage of proteins was calculated with DichroWeb website’s http://dichroweb.cryst.bbk.ac.uk/html/home.shtml (accessed on 1 September 2024) K2d method [12]. All samples with a concentration of 0.0215 mg/mL were scanned at a heating rate of 5 °C min^−1^ from 10 to 100 °C.

### 2.8. Field Emission Scanning Electron Microscopy (FE-SEM)

The morphology of the proteins was examined using a scanning electron microscope (JEOL JSM 7800F, Tokyo, Japan). Four freeze-dried samples—mantle (control), sarcoplasmic, stromal, and myofibrillar proteins—were mounted on a carbon grid and placed under vacuum. Images were collected at an accelerating voltage of 1 kV. The results are presented as micrographs [13].

## 3. Results and Discussions

### 3.1. Fourier-Transform Infrared Spectroscopy (FT-IR)

**Figure 1:**
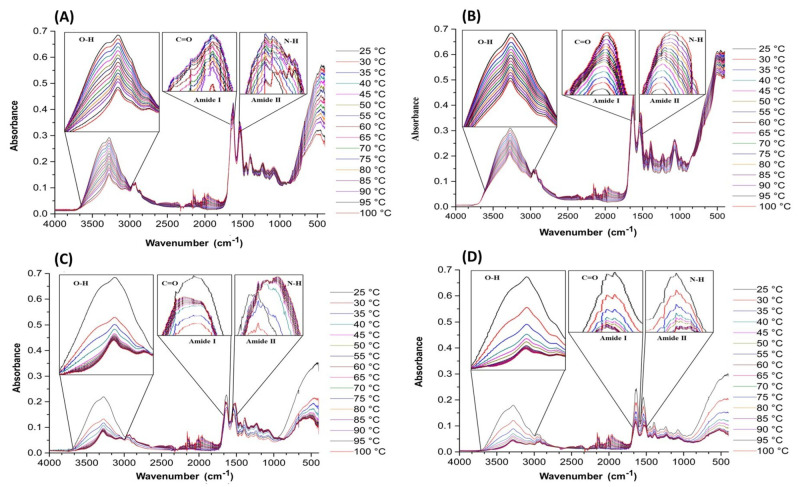
The effect of temperature on the infrared spectrum of the giant squid (*Dosidicus gigas*): (**A**) Mantle, (**B**) sarcoplasmic, (**C**) myofibrillary, and (**D**) stromal.

Sixteen FT-IR spectra were obtained for each protein fraction across the 25–100 °C temperature range. Figure 1A displays the overall spectral evolution of proteins (sarcoplasmic, myofibrillar, and stromal) from the *D. gigas* mantle, where you can see gradual variations in their structure. Specifically, alterations are observed in the amide I band at 1600 cm^−1^ (C=O), which are related to modifications in the secondary structure of proteins [14]. The FT-IR spectra for sarcoplasmic proteins (Figure 1B) showed significant changes in the absorption band of the carbonyl group (C=O), which corresponds to the amide I region and is located around 1600 cm^−1^. As can be seen, the intensity and amplitude of the absorption band are affected. This is consistent with previous research on these proteins, as documented by Yongsawatdigul and Hemung [15]. Furthermore, modifications in hydroxyl (-OH) groups are also discerned, which arise at around 3300 cm^−1^ in the spectra, pointing towards important changes in molecular interactions [16]. On the other hand, myofibrillar (1C) and stromal proteins (1D) show significant changes in absorbance with temperature variations. Stromal proteins, which are rich in collagen, showed a marked decrease in absorbance from 25 to 50 °C, reflecting the progressive disruption of hydrogen bonds that stabilize the collagen triple helix. This early loss of structural integrity aligns with the known thermal lability of collagen in connective tissues. Myofibrillar proteins showed an intermediate response: a moderate but consistent decrease in absorbance, indicating unfolding of α-helical domains in proteins like myosin and actin. These differences in thermal sensitivity observed at 3300 cm^−1^ highlight the distinct molecular organization and hydrogen-bonding patterns among the protein fractions and underscore the structural rigidity of connective tissue proteins compared to the more dynamic nature of myofibrillar proteins.

**Figure 2:**
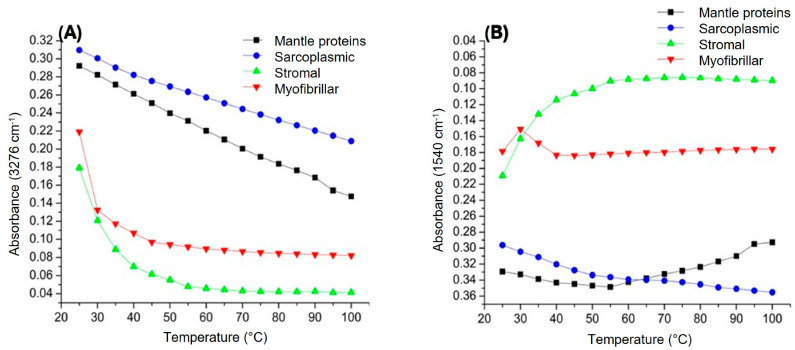
Effect of temperature on the infrared spectrum of giant squid (*Dosidicus gigas*). (**A**) Groups NH (3276 cm^−1^) and (**B**) amide II (1540 cm^−1^).

Figure 2A shows the vibration of the NH stretch at 3276 cm^−1^ (amide A), while Figure 2B illustrates the behavior of amide II (1540 cm^−1^), which is related to the out-of-phase combination of the NH in plane bend and the CN-stretching vibration with less contribution from the CO bend in the plane, as well as CC- and NC-stretching vibrations. In proteins, the absorption at 3276 cm^−1^ is attributed to the NH group and is insensitive to the conformation of the polypeptide backbone. All these bonds are those involved in the conformation of the peptide bond; for this reason, changes in their absorbance with respect to temperature are indicative of protein stability [6]. Regarding sarcoplasmic and mantle proteins, Figure 2A shows a gradual decrease in both groups of proteins as the temperature increases. This could be due to the fact that, as the temperature increases, the hydrogen bonds that stabilize secondary structures (such as α-helices and β-sheets) can be broken, thus reducing the intensity of the band. On the other hand, when the amide II of the mantle proteins was studied, it can be noticed that absorbance at 1540 cm^−1^ decrease from 25 to 50 °C and then increase from 55 to 100 °C; this behavior indicates a dynamic equilibrium in the interactions of the amide groups with their environment, which can be due to a dynamic interplay between hydrogen bond disruption and formation of new dipolar interactions. Between 25 and 55 °C, hydrogen bond breaking and the increased molecular disorder reduce the intensity. However, between 55 and 100 °C, the formation of new interactions or structural reorganization (such as aggregates or denatured structures) can stabilize the amide group, again increasing the intensity [17]. Meanwhile, sarcoplasmic protein showed a decrease throughout the temperature range studied, which suggests a gradual thermal denaturation process, characterized by the progressive loss of its tertiary and secondary structure, hydrogen bond stabilizer disruption, and the possible formation of aggregates [18,19]. Interestingly, both sarcoplasmic proteins and mantle proteins exhibited comparable patterns in this region, indicating that sarcoplasmic components may heavily influence the overall thermal response of the mantle’s protein matrix in this specific vibration mode. This could be due to the relatively high proportion and flexibility of sarcoplasmic proteins, which may dominate the spectroscopic signal in this region or drive cooperative unfolding–reorganization events upon heating. The distinct behavior of this band also suggests that Amide II may serve as a sensitive marker for subtle conformational transitions, especially in less ordered or more mobile protein fractions.

In relation to stromal proteins, a greater effect was found between 25 and 60 °C. As in the rest of the proteins, the decrease in intensity at 3276 cm^−1^ can be attributed to the breaking of hydrogen bonds as a consequence of an increase in temperature, reducing the intensity of the signal associated with the stretching of NH bond, since these bonds play a crucial role in the stabilization of collagen triple helix (main component of stromal proteins). When the absorption of the band at 1540 cm^−1^ is monitored, a similar but inverse behavior is observed, that is, an abrupt increase in the intensity of the band at 1540 cm^−1^ (25–60 °C), and then it remains almost unchanged after 60 °C. The increase could be attributed to a molecular reorganization, favoring intramolecular and dipolar interactions, especially those related to NH and CN bonds. Likewise, in this temperature range, the breakdown of weak interactions is favored, leading to greater exposure of amide groups and increasing their contribution to the dipole moment during vibration, intensifying the band. Subsequently, after 60 °C, this group of proteins seems to reach a structural state in which no additional significant changes occur that affect the dipolar interactions or the vibrational dynamics of the NH and CN bonds [14]. It is important to note that marine-derived collagens, such as those found in squid stromal proteins, typically exhibit lower denaturation temperatures compared to collagens from terrestrial animals, due to differences in amino acid composition and lower hydroxyproline content [20].

The effect of temperature on myofibrillar proteins showed a similar behavior to that of stromal proteins, that is, an abrupt decrease between 25 and 50 °C for amide A (3276 cm^−1^), and then remaining almost unchanged. This suggests the breaking of hydrogen bonds that stabilize the secondary structure, being a typical behavior in this temperature range, which is associated with thermal denaturation. This leads to the loss of ordered secondary structures, exposing NH groups to the medium [21,22]. Regarding the absorption at 1540 cm^−1^ (amide II), an absorption peak was detected between 25 and 40 °C and then remained almost constant. It could be associated with the denaturation of this protein fraction, this can be due to an increase in molecular mobility, facilitating the formation or strengthening of dipolar interactions related to NH and CN bonds, intensifying the absorption of the band, while from 40 to 100 °C, a thermal and structural equilibrium is reached where no further significant changes occur that affect the absorption of the amide II band [23].

**Figure 3:**
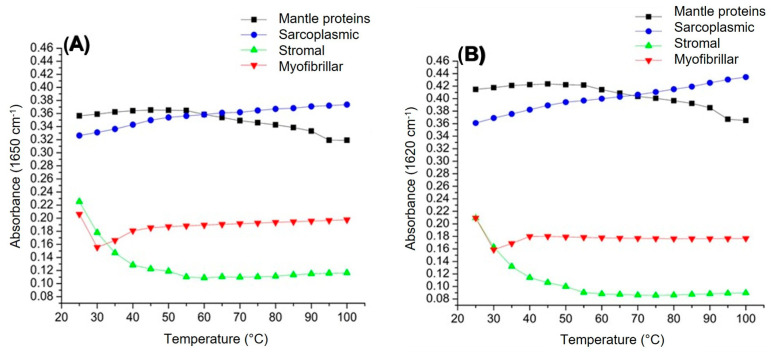
Effect of temperature on the infrared spectrum of the giant squid (*Dosidicus gigas*). (**A**) Disordered structure-1650 cm^−1^ and (**B**) ordered structure 1620 cm^−1^.

Figure 3 illustrates the intensities of the vibrational modes associated with Amide I at 1650 cm^−1^ and 1620 cm^−1^. These vibrations primarily arise from the C=O stretching, with smaller contributions from out-of-phase CN stretching, CCN deformation, and NH in-plane bending. Since these vibrations are closely linked to the secondary structure of the protein backbone, amide I is the most frequently analyzed region for secondary structure studies. The intensity of the band at 1650 cm^−1^ is related to the α-helix structure, while the band at 1620 cm^−1^ is frequently associated with β-sheets [6,14].

Regarding these absorption bands, the squid mantle proteins showed a similar behavior, that is, an increase from 25 to 55 °C followed by a decrease (55–100 °C). This initial increase could be attributed to thermal structural changes caused by the breaking of hydrogen bonds and other intra- and intermolecular interactions; that is, proteins lose their native conformation. This unfolding leads to the formation of ordered structures, such as alpha-helical and beta sheets. The subsequent decrease suggests thermal denaturation of this structure, possibly accompanied by protein aggregation [24]. On the other hand, sarcoplasmic proteins show a gradual increase in the intensity of both bands (1650 and 1620 cm^−1^) as the temperature increases. At low temperatures, this could suggest protein unfolding, but the gradual increase at higher temperatures can be attributed to the increase in the population of excited vibrational states and greater molecular mobility that facilitates vibrations, rather than being directly related to changes in secondary structure [25]. Regarding the stromal proteins, a similar behavior at 1650 cm^−1^ and 1620 cm^−1^ was observed; they presented a sharp decrease from 25 to 55 °C, and then they remained almost constant up to 100 °C. In this sense, the absorbance reduction at low temperatures can be attributed to the denaturation process, and it can be associated with the rupture of non-covalent bonds between proteins, which, in turn, leads to secondary structure losses. This behavior has been reported by Veeruraj et al. [26], indicating a denaturation temperature for isolated collagen between 35 and 38 °C. Similarly, myofibrillar proteins showed similar behavior (1650 and 1620 cm^−1^): a decrease was found between 25 and 40 °C, and then they remained almost unchanged. This decline suggests an early loss of ordered secondary structures, likely due to the disruption of hydrogen bonds and weakening of stabilizing intramolecular interactions. The simultaneous reduction in both α-helix and β-sheet signals implies that the protein matrix undergoes an unfolding process [25]. Beyond 40 °C, the absorbance values for both bands remained relatively stable, indicating that the major conformational changes occurred within the lower temperature range. This behavior may reflect the completion of thermal denaturation events in this fraction, leading to an equilibrium state where additional temperature increments no longer significantly affect the vibrational modes associated with these secondary structures.

### 3.2. Circular Dichroism (CD)

**Figure 4:**
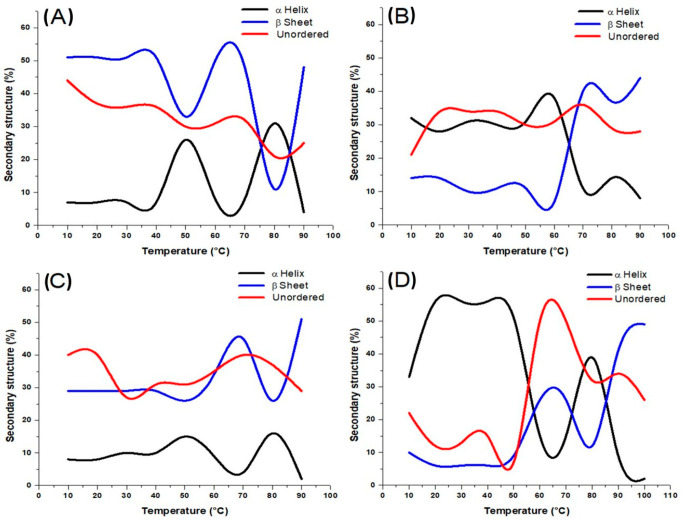
(**A**) Secondary structure content of sarcoplasmic proteins. (**B**) Secondary structure content of myofibrillar proteins. (**C**) Secondary structure content of stromal proteins. (**D**) Secondary structure content of mantle proteins.

When protein solutions are heated to reach their denaturation temperature, molecules gain kinetic energy, resulting in significant electrostatic repulsion [7,27], which can affect the β-sheets and α-helices, transforming them into a random configuration. Figure 4 shows the secondary structure content of sarcoplasmic (A), myofibrillar (B), stromal proteins (C), and mantle proteins from the giant squid (D) as the temperature increases. It is noticed that there is an inversely proportional relationship between α-helix and β-sheet structures in all the protein systems. As can be seen (Figure 4A), at low and high temperatures, β-sheet and unordered structures are the predominant for sarcoplasmic proteins, while at 50 and 80 °C, α-helix structures showed their maximum peaks. This suggests that in native conditions, sarcoplasmic proteins from *Dosidicus gigas* are characterized by a flexible and disordered conformation, which is typical for water-soluble proteins with low structural compactness [28]. As the temperature increased to 50 °C and 80 °C, the α-helix content showed notable peaks, indicating partial refolding or structural rearrangement of polypeptides into more ordered helical conformations. This intermediate stabilization may be attributed to increased molecular mobility and unfolding of less stable structures (such as random coils or turns), allowing for the transient formation of α-helical regions stabilized by newly formed hydrogen bonds. This phenomenon is often associated with molten globule-like states, where proteins retain some secondary structure but lack defined tertiary organization [25,29].

The secondary structure of myofibrillar proteins from *Dosidicus gigas* showed significant thermal sensitivity (Figure 4B). At low temperatures, α-helix structures predominated, consistent with the native conformation of structural proteins like myosin and actin. As the temperature increased to 55 °C, myofibrillar proteins from *Dosidicus gigas* exhibited an increase in α-helix content, alongside a decrease in β-sheet and unordered structures. This suggests a heat-induced structural rearrangement, where previously disordered or partially unfolded regions may refold into α-helical conformations. The reduction in β-sheets indicates a reversible conformational shift rather than progressive denaturation. This transition may reflect a molten globule-like intermediate state, temporarily stabilizing protein structure. Such behavior is relevant for food processing, as it highlights a temperature range where functional properties like gelation or network formation can be optimized through controlled thermal treatment. Between 60 and 95 °C, myofibrillar proteins exhibited a progressive increase in β-sheet structures, which become the predominant secondary conformation, while α-helix content continues to decline, reaching its lowest point. Such behavior is typical of heat-induced aggregation, where β-structures dominate due to intermolecular hydrogen bonding. This structural transition is crucial for understanding the textural changes and gelation behavior of myofibrillar proteins during thermal processing [30].

The thermal behavior of stromal proteins showed a distinct response compared to other muscle protein fractions (Figure 4C). At low temperatures, unordered and β-sheet structures were predominant, with lower proportions of α-helix content, reflecting the inherently disordered or aggregated nature of collagen-rich stromal proteins. As the temperature increased to 45–50 °C, a slight rise in α-helix content was observed, likely indicating a transient structural rearrangement or partial stabilization of helical regions. However, this was short-lived. From 65 to 75 °C, α-helix content declined sharply, while β-sheet structures increased significantly, becoming the dominant secondary structure above 70 °C. This shift reflects irreversible thermal denaturation and the formation of stable intermolecular β-aggregates, which are commonly observed in collagen and gelatin systems upon heating [25]. Unordered structures also increased moderately, supporting the notion of disrupted triple-helix organization and transition to amorphous or aggregated states. At 95 °C, stromal proteins exhibited a profile dominated by β-sheet and unordered conformations, confirming extensive structural disruption. These changes are relevant for the development of biomaterials or functional ingredients from squid stromal proteins, where controlled thermal treatment could modulate aggregation and network formation for desired mechanical or rheological properties.

The secondary structure evolution of mantle proteins, comprising myofibrillar, sarcoplasmic, and stromal proteins, revealed complex thermal behavior due to the heterogeneous nature of the system (Figure 4D). At low temperatures, the α-helix was the dominant structure, reflecting the high proportion of myofibrillar proteins in the mixture. Between 60 and 95 °C, β-sheet and unordered structures progressively increased, with β-sheets becoming predominant at higher temperatures. This transition indicates thermal denaturation and the formation of intermolecular β-aggregates, driven by protein unfolding and exposure of hydrophobic residues [31]. The presence of sarcoplasmic and stromal proteins may also contribute to the unordered fraction, especially as they are more susceptible to structural disruption [32]. Overall, the mantle proteins exhibit intermediate behavior between their major components, dominated by myofibrillar protein transitions but modulated by the contributions of the other fractions. These results highlight the importance of protein interactions and composition in determining thermal responses, which is relevant for the development of squid-based products with optimized texture and stability.

### 3.3. Field Emission Scanning Electron Microscopy (FE-SEM)

**Figure 5:**
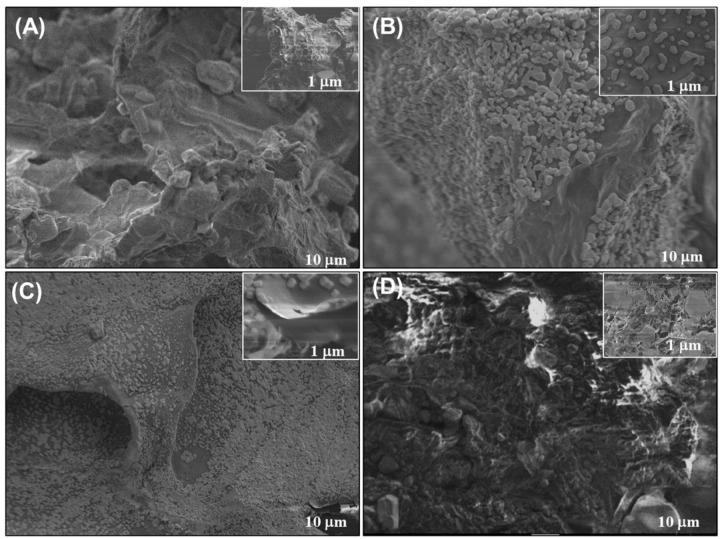
FE-SEM micrographs of giant squid (*Dosidicus gigas*) mantle proteins: (**A**) mantle, (**B**) sarcoplasmic, (**C**) myofibrillar, and (**D**) stromal.

Field emission scanning electron microscopy (FE-SEM) provided complementary insights into the thermal response of squid mantle proteins (Figure 5). Each protein fraction exhibited distinctive microstructural features. Total protein mix (Figure 5A) exhibited a compact, heterogeneous matrix with irregular aggregates and partial porosity, indicative of interactions among globular and fibrous proteins forming a dense network. Comparable observations were detected in prior research involving Dosidicus gigas, in which proteins created voids separated by thin layers and interweaving of proteins in the unprocessed mantle [5,13,33]. While thin layers or walls were visible, these may be attributed to higher molecular weight proteins, such as myosin. Sarcoplasmic proteins (Figure 5B) displayed predominantly smooth, amorphous, and featureless surfaces, consistent with their water-soluble, globular nature and lack of fibrous assembly. These proteins have low molecular weights ranging from 66 to 36 kDa, which makes them smaller in size at 10 μm, in comparison to myofibrillar and stromal proteins, with molecular weights of 200 kDa and 170–180 kDa, respectively, as documented in previous research [34,35]. Their globular shape tends to create agglomerates, as shown by the micrograph with areas of protein fusion forming less defined structures [6,14]. Myofibrillar proteins (Figure 5C) revealed well-defined, interwoven filamentous networks with porous regions, characteristic of actin–myosin structures. Such architecture aligns with their ability to form thermo-stable gels and emulsions [3,5]. Stromal proteins (Figure 5D) exhibited rough, dense, and layered surfaces, which is a characteristic of stromal proteins like collagen and elastin [13]. Similar findings were reported by Sun et al. [36], who attribute the fibrous shape to the triple helix structure of collagen from D. Gigas. These FE SEM observations underscore that each protein fraction from squid mantle has a unique microstructural shape related closely to its solubility, structural composition, and functional behavior in gel formation, emulsification, or biomaterial development. It is important to note that the FE-SEM micrographs presented in this study correspond to the raw, non-heated protein isolates. While thermal treatment is known to induce significant morphological changes in protein matrices, it was not possible to include micrographs of heat-treated samples due to logistical limitations. Despite this, the micrographs of the untreated protein fractions still provide relevant insights into their inherent morphological characteristics, particularly when interpreted alongside the FT-IR and CD data. This integrative approach enables a comprehensive understanding of how structural composition relates to thermal behavior, even in the absence of thermally modified visual evidence.

## 4. Conclusions

This study provides novel insights into the thermal behavior of individual muscle protein fractions (sarcoplasmic, myofibrillar, and stromal) from Dosidicus gigas by combining FT-IR, circular dichroism (CD), and FE-SEM techniques. Our results highlight distinct temperature-dependent structural transitions in each protein class. FT-IR revealed that stromal proteins, rich in marine collagen, undergo early denaturation and hydrogen bond disruption at lower temperatures compared to other fractions, confirming their thermal sensitivity. The CD analysis uniquely revealed the stabilization of intermediate α-helix structures at specific temperatures in sarcoplasmic and myofibrillar proteins, which was indicative of molten globule-like states that are rarely reported in cephalopod proteins. Furthermore, despite not including heat-treated samples, FE-SEM micrographs supported the compositional and morphological differentiation among fractions, aligning with their unfolding and aggregation observed in spectroscopic data. These findings contribute to a better understanding of the structural behavior of squid muscle proteins, supporting their potential use in functional foods, gels, or bio-based materials.

## Data Availability

The original contributions presented in this study are included in the article. Further inquiries can be directed to the corresponding author.
